# Lessons From the Real World: Preparing Dementia Care Partners Using REACH-TX

**DOI:** 10.1093/geroni/igaf122.238

**Published:** 2025-12-31

**Authors:** Stephani Shivers, Dante Tipiani, Amy Puca, Vicki Zimmer, Alan Stevens, Edward Cisek

**Affiliations:** CaringKind, New York, New York, United States; CaringKind, New York, New York, United States; Via Evaluation, Buffalo, New York, United States; Via Evaluation, Buffalo, New York, United States; Baylor Scott & White Health, Belton, Texas, United States; CaringKind, New York, New York, United States

## Abstract

Community-based organizations (CBO) are strongly positioned to translate evidence-based interventions into real world practice. CaringKind, a dementia focused CBO, examined the effects of implementing Resources for Enhancing Alzheimer’s Caregiver Health-II (REACH)-TX in a project funded by the Administration on Community Living. REACH-II is a widely studied, evidence-based education/support program for dementia family care partners (CP). The REACH-TX version of the program engages a CP with a certified REACH specialist for 2-6 meetings over the course of 3-6 months. REACH-TX includes a risk assessment, tailored plan of care, and skills-training activities focusing on 9 core areas including safety, promoting emotional well-being, and managing problem behaviors. Real world adaptations included virtual delivery, elimination of in-home visits, and an internal staff master trainer. To practically evaluate the impact of REACH-TX, CaringKind chose the Preparedness for Caregiving Scale (PCGS) as the one measure to be completed by CP at baseline and program completion. Results indicated a significant increase in PCGS scores from 2.1 to 2.8 (t(64)=6.12, p<.001; possible range 0-4). Overall, 75% of CP had an increase in their mean PCGS score. Over half of the 57 CP who answered an added question about confidence in their ability to handle challenging or distressing behaviors (31, 54%) reported a higher score post intervention. This pragmatic delivery of REACH-TX by a CBO demonstrated a positive outcome on the PCGS, which aligns with the intervention and is realistic to administer. Leveraging CBOs in intervention development, translation and implementation may accelerate intervention uptake and adoption.

